# Masse rétro péritonéale kystique et multi-cloisonnée: kyste hydatique ou tumeur nerveuse?

**DOI:** 10.11604/pamj.2014.19.131.5488

**Published:** 2014-10-06

**Authors:** Pierlesky Elion Ossibi, Imane Kamaoui

**Affiliations:** 1 Service de Chirurgie Viscérale B, CHU Hassan II, Fès, Maroc; 2 Service de Radiologie, CHU Hassan II, Fès, Maroc

**Keywords:** Péritonéale kystique, kyste hydatique, canalaire, cystic peritonitis, hydatid cyst, ductal

## Image en medicine

Les masses retro-péritonéales kystiques et multi-cloisonnées sont rares. Plusieurs diagnostics peuvent être évoqués devant une masse retro-péritonéale kystique et multi-cloisonnée parmi lesquels: un kyste hydatique du psoas ou une tumeur nerveuse. Nous rapportons l'observation d'une patiente âgée de 50 ans sans antécédent pathologique notable qui présente depuis 6 mois des douleurs isolées du flanc gauche. L'examen clinique trouve une patiente en bon état général avec une légère sensibilité à la palpation du flanc gauche. L'examen neurologique est normal. Le bilan biologique est normal. L’échographie abdominale est revenue en faveur d'un abcès froid ou d'un kyste hydatique du psoas gauche. Le scanner abdominal montre une masse rétropéritonéale gauche avec une composante endo canalaire et exo canalaire. Elle est de forme ovalaire majoritairement kystique, contenant de multiples cloisons épaisses et mesure 90 x 117 mm. S'agit-il d'une tumeur neurogène ou d'un kyste hydatique (A et B). Le complément IRM lombaire a mis en évidence cette volumineuse masse, bien limitée, ovalaire, multi-loculée, se rehaussant de manière hétérogène, délimitant des zones liquéfiées qui mesure 86 x 92 x 120 mm. Elle s’étend en dedans en endo canalaire, élargissant le foramen gauche de L1 et venant comprimer le cône médullaire et dissocier les racines nerveuses (C et D). La prise de contraste n'est pas en faveur d'un kyste hydatique. La patiente est adressée en neurochirurgie pour une prise en charge.

**Figure 1:**
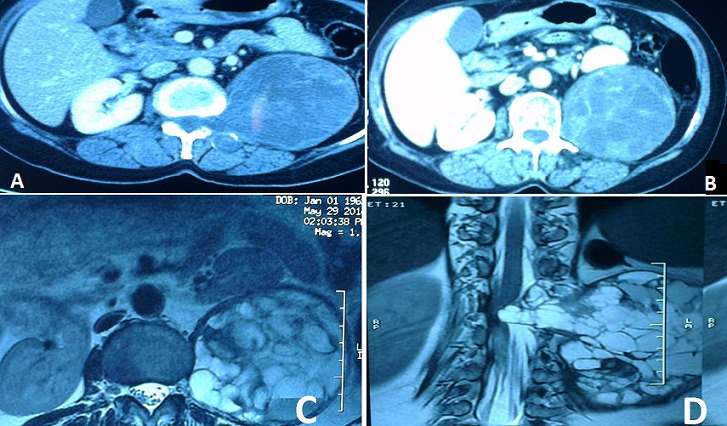
(A) scannographie montrant la volumineuse masse rétro-péritonéale hétérogène; (B) discret rehaussement de la masse après injection de produit de contraste; (C) IRM de la masse rétro-péritonéale multi-cloisonnée; (D) masse élargissant le foramen gauche de L1 avec compression médullaire

